# Comparison Between Clinicopathological Characteristics, *BRAF* V600E and *TERT* Promoter Mutation of Familial Non-Medullary Thyroid Carcinomas, and Sporadic Case

**DOI:** 10.3389/fonc.2021.616974

**Published:** 2021-12-01

**Authors:** Tian Yang, Longsheng Huang, Chang Chen, Han Luo, Yong Jiang

**Affiliations:** ^1^ Department of Pathology, West China Hospital of Sichuan University, Chengdu, China; ^2^ West China School of Medicine, Sichuan University, Chengdu, China; ^3^ Thyroid and Parathyroid Surgery Center, West China Hospital of Sichuan University, Chengdu, China

**Keywords:** familial non-medullary thyroid carcinomas, clinicopathological characteristics, *BRAF* V600E, *TERT* promoter, papillary thyroid cancer

## Abstract

**Background:**

It has been debated whether familial non-medullary thyroid carcinoma (FNMTC) is more aggressive and has a worse prognosis than sporadic non-medullary thyroid carcinoma (SNMTC). Our aim was to compare the invasiveness and prognosis of FNMTC and SNMTC by their biological behavior and molecular changes.

**Method and Material:**

Our group mainly compared 106 patients with FNMTC whom have complete clinicopathological data during 2011–2019 in West China Hospital, Sichuan University, and 212 randomly selected cases with SNMTC were included to compare their biological behavior, recurrence and mortality, and molecular expression of *BRAF* V600E and *TERT* promoter. At the same time, FNMTC cases were divided into four subgroups, namely, two affected members group, three or more affected members, parent/offspring group, and sibling group, and they were compared with SNMTC separately to analyze the difference in their invasiveness and prognosis.

**Results:**

We found that the mean tumor size of FNMTC (0.96 ± 0.53cm) was smaller than that of SNMTC (1.15 ± 0.72 cm) (*p* = 0.020), while no significant difference in the incidence of other clinicopathological factors, including bilateral growth, capsular invasion, with thyroid nodular goiter or not, multifocality, lymph node metastasis, extrathyroidal extension, iodine 131 treatments, T stage, and American Joint Committee on Cancer (AJCC) stage, was observed between FNMTC and SNMTC (*p* > 0.05), between each FNMTC subgroup (*p* > 0.05), and between each FNMTC subgroup and SNMTC (*p >* 0.05). There was no significant difference in recurrence, mortality, and *BRAF* V600E and *TERT* promoter mutation between FNMTC and SNMTC, among which 50/60 (83.33%) of FNMTC patients had *BRAF* V600E mutation and 1/32 (3.13%) had *TERT* promoter mutation, while the mutation rates of SNMTC were 93/108 (86.11%) and 3/64 (4.69%) (*p* > 0.05).

**Conclusion:**

There was no significant difference in invasiveness and prognosis between FNMTC and SNMTC by biological behavior, patient survival, and molecular level comparison.

## Introduction

Due to the increased application of ultrasound and thyroid fine needle aspiration (FNA) biopsy, thyroid cancer (TC), a tumor originating from the thyroid follicular or parafollicular epithelium, has an increasing incidence and becomes the most common endocrine malignant tumor (1–3). Non-medullary thyroid carcinoma (NMTC) arises from thyroid follicular epithelial cells and includes papillary, follicular, and undifferentiated carcinomas, accounting for 95% of TC. NMTC, which mostly occurs in a sporadic fashion, usually has less aggressive behavior and a good prognosis (4).

Familial non-medullary thyroid carcinoma (FNMTC), consisting of 5–10% of thyroid carcinoma, is defined as NMTC occurring in two or more first-degree relatives in the absence of other predisposing causes of thyroid cancer (3–6). There has been debates about the difference in aggressiveness between FNMTC and sporadic non-medullary thyroid carcinoma (SNMTC). Several studies reported that FNMTC tended to be more aggressive than SNMTC, and FNMTC had increased risk of multifocal, bilateral growth, capsular and vascular invasion, and lymph node metastasis, with relatively short disease-free survival (5, 7–10). The patients with three or more cases in a family were more aggressive and had a poorer prognosis than those with only two cases in a family (7), and parent–offspring FNMTC might have more aggressive biological behavior and a worse prognosis than sibling FNMTC (11). However, there were other studies indicated that FNMTC was no more aggressive than SNMTC (12–15).

v-Raf murine sarcoma viral oncogene homolog B1 (*BRAF*) V600E mutation is the most common genetic mutation in papillary thyroid carcinoma (PTC); it is associated with thyroid cancer by activating the mitogen-activated protein kinase pathway (16). Telomerase reverse transcriptase (*TERT*) is the catalytic subunit of telomerase. The two most common mutations in the *TERT* promoter region are C228T and C250T, which can enhance the transcriptional activity of the *TERT* promoter and are frequently found in TC (17). At present, some studies have performed combined analysis of *BRAF* V600E mutation and *TERT* promoter mutation in NMTC and found that these mutations were significantly related to the pathogenesis, development, and prognosis of NMTC (18, 19). Some studies compared the type of common gene mutations, including *BRAF*, *Ras*, between FNMTC and SNMTC, and found no significant differences (13).

To clarify the clinicopathological characteristics of FNMTC and compare the tumor aggressiveness between these two malignancies, we retrospectively analyzed the PTC patients who underwent thyroid FNA in West China Hospital. 

## Method and Material

We reviewed 177 patients from 80 families who underwent thyroid FNA and were diagnosed with NMTC in West China Hospital, Sichuan University, during 2011–2019. Those patients including 175 PTC and 2 follicular carcinoma had no other predisposing causes of thyroid cancer and had been classified as FNMTC according to criteria defined by Sturgeon (20).

However, only 106 patients with postoperative pathology diagnosis of PTC were included in this study due to the incomplete clinicopathological data caused by the fact that some patients did not receive surgery in our hospital (including 5 patients who did not receive surgery, and the rest were operated on in other hospitals), and some patients were lost to follow-up. A total of 7,663 patients were diagnosed with PTC in our hospital records system, and 212 cases were randomly selected to match 1:2 ratio for study purpose. All of those patients’ negative family history of thyroid carcinoma was confirmed during the telephone follow-up. The mean follow-up time for FNMTC patients was 42 months (6–120 months) and that for SNMTC was 44.5 months (7–102 months).

T (the extent of the primary tumor), N (regional lymph-node metastases), and M (distant metastases) staging was determined according to the eighth edition of the American Joint Committee on Cancer (AJCC) thyroid cancer staging criteria.

DNA from paraffin-embedded tumor tissues (all samples came from preoperative FNA cell blocks or postoperative tumor tissue paraffin blocks) was extracted according to the instructions of QIAamp DNA FFPE Tissue Kit from QIAGEN, Germany. The fC-1100 ultrafine ultraviolet spectrophotometer produced by Hangzhou Suizhen Co., Ltd. was used to detect its purity and concentration and stored at −20°C for future use.

Polymerase chain reaction (PCR) was used for amplification. For *BRAF* V600E, PCR primers were used to amplify the exon 15 of *BRAF* gene containing mutation hotspots (16). For *TERT* promoter mutations, the previously established PCR primers 50-AGTGGATTCGCGGGCACAGA-30 (sense) and 50-CAGCGCTGCCTGAAACTC-30 (antisense) were used to amplify the *TERT* promoters containing two mutation hotspots (C228T and C250T) (17). These materials were carried out with an initial denaturation step at 95°C for 3 min, followed by 10 cycles of 95°C denaturation for 30 s, 55°C annealing for 30s, and 68°C elongation for 1 min. Before sequencing, 20 g/L agarose gel electrophoresis was used to detect the quality of PCR amplification products, and Sanger sequencing was performed on PCR amplification products with satisfactory quality. The sequencing results were compared with the *BRAF* and *TERT* gene sequences to confirm the mutation status.

### Statistical Method

All data were analyzed by the Statistical Package for the Social Sciences 21.0 (SPSS Inc., Chicago, IL, USA). Chi-square test or Fisher’s exact test were used to compare differences in the intergroup count data, and t-test or Mann-Whitney U test was used to compare differences in the intergroup count data; *p* < 0.05 were considered statistical significant.

### Ethical Approval

All procedures in this study were reviewed and approved by the Clinical Trial and Biomedical Ethics Committee of West China Hospital of Sichuan University. Informed consent in this retrospective study is excused. 

## Results

### Clinicopathological Features of FNMTC Patients and SNMTC

We collected 177 FNMTC patients from 80 families, and 106 of them were operated on in West China Hospital, Sichuan University, with a complete clinical and pathological data. The median age at diagnosis was 44.2 ± 12.1 years (range, 7–72 years) and 41.7 ± 11.65 years (range, 12–83 years), respectively, for FNMTC and SNMTC patients (*p* = 0.078). There were 23 (21.70%) male patients and 83 (78.30%) female patients, with the M:F ratio of 1:3.6, while in the control group, 56 (26.42%) were male and 156 (73.58%) are female, with a M/F ratio of 1/2.8 (*p* = 0.359) (**Table 1**). The mean tumor sizes of FNMTC was 0.96 ± 0.53 cm (range, 0.2–3.7 cm), smaller than that of SNMTC (1.15 ± 0.72 cm; range, 0.1–5.0 cm) (*p* = 0.020).

**Table 1:** Clinicopathological characteristics and prognostic factors of FNMTC versus SNMTC, n (%).

Parameter	FNMTC^a^	SNMTC^b^	*p*
	(n = 106)	(n = 212)	
Gender			
Male	23 (21.70)	56 (26.42)	0.359
Female	83 (78.30)	156 (73.58)	
Age			
<55 years	87 (82.08)	186 (87.74)	0.172
≥55 years	19 (17.92)	26 (12.26)	
Tumor size (cm)			
≤1.0	76 (71.70)	125 (58.96)	0.026
>1.0	30 (28.30)	87 (41.04)	
Capsular invasion			
Yes	74 (69.81)	155 (73.11)	0.536
No	32 (30.19)	57 (26.89)	
Multifocality			
Yes	33 (31.13)	74 (34.91)	0.502
No	73 (68.87)	138 (65.09)	
Bilaterality			
Bilateral	22 (20.75)	48 (22.64)	0.702
Unilateral	84 (79.25)	164 (77.36)	
With HT^c^			
Yes	14 (13.21)	46 (21.70)	0.068
No	92 (86.79)	166 (78.30)	
Thyroid nodular goiter			
Yes	63 (59.43)	114 (53.77)	0.338
No	43 (40.57)	98 (46.23)	
Lymph node metastasis			
Yes	66 (62.26)	136 (64.15)	0.511
No	40 (37.74)	70 (33.02)	
ETE^d^			
Yes	17 (16.04)	48 (22.64)	0.169
No	89 (83.96)	164 (77.36)	
Treatment of iodine-131			
Treated	30 (28.30)	78 (36.79)	0.057
Untreated	54 (50.94)	83 (39.15)	
Missing data	22 (20.76)	51 (24.06)	
T stage			
T1 + T2	95 (89.62)	186 (87.74)	0.621
T3 + T4	11 (10.38)	26 (12.26)	
AJCC stage			
I + II	104 (98.11)	208 (98.11)	NS^e^
III + IV	2 (1.89)	4 (1.89)	

a FNMTC, familial non-medullary thyroid carcinoma.

b SNMTC, sporadic non-medullary thyroid carcinoma.

c HT, Hashimoto thyroiditis.

d ETE, extrathyroidal extension.

e NS, not significant.

We compared the clinicopathological features of FNMTC and SNMTC and found that the tumor size of FNMTC was significantly smaller than SNMTC (*p* = 0.026), while no significant difference in the incidence of other clinicopathological factors was observed between the two groups, including bilateral growth, capsular invasion, with Hashimoto thyroiditis (HT) or thyroid nodular goiter or not, multifocality, lymph node metastasis, extrathyroidal extension, iodine 131 treatments, T stage, and AJCC stage (*p* > 0.05) (**Table 1**).

### Recurrence and Mortality of FNMTC and SNMTC

We collected 140 patients’ follow-up data, including 106 cases that we analyzed and one follicular carcinoma patient, from 177 FNMTC patients. There were four (2.86%) relapsed and one (0.71%) deceased from FNMTC, while nine (4.25%) relapsed but no deceased from SNMTC. There were no significant difference in recurrence rate and mortality between the two groups (*p* > 0.05) (**Table 2**).

**Table 2:** Recurrence and mortality of FNMTC versus SNMTC, n (%).

Parameter	FNMTC^a^	SNMTC^b^	*p*
	(n = 140)	(n = 212)	
Recurrence			
Yes	4 (2.86)	9 (4.25)	0.447
No	136 (97.14)	193 (91.04)	
Death from disease			
Yes	1 (0.71)	0 (0)	NS^c^
No	139(99.29)	212 (100)	

a FNMTC, familial non-medullary thyroid carcinoma.

b SNMTC, sporadic non-medullary thyroid carcinoma.

c NS, not significant.

### Clinicopathological Features of FNMTC Subgroup Patients and SNMTC

There were 92 (86.79%) patients of FNMTC who came from two affected members families and 14 (13.21%) from three or more affected families. Compared with the two affected members subgroup, SNMTC group has an increased incidence of Hashimoto thyroiditis (*p* = 0.046). However, no significant difference was observed between the group of two affected FNMTC, three or more affected FNMTC, and SNMTC in other clinicopathological factors, including gender, age, tumor size, capsular invasion, bilateral growth, with thyroid nodular goiter or not, multifocality, lymph node metastasis, extrathyroidal extension, iodine 131 treatments, T stage, and AJCC stage (*p* > 0.05) (**Table 3**).

**Table 3:** Clinicopathological characteristics and prognostic factors of two affected members group and three or more affected members group vs. SNMTC, n (%).

Parameter	Two affected members	Three or more affected members	p	SNMTC^a^	*p*a^b^	*p*b^c^
	(n = 92)	(n = 14)		(n = 212)		
Gender						
Male	22 (23.91)	1 (7.14)	0.294	56 (26.42)	0.646	0.199
Female	70 (76.09)	13 (92.86)		156 (73.58)		
Age						
<55 years	74 (80.43)	13 (92.86)	0.456	186 (87.74)	0.096	NS
≥55 years	18 (19.57)	1 (7.14)		26 (12.26)		
Tumor size (cm)						
≤1.0	65 (70.65)	11 (78.57)	0.456	125 (58.96)	0.053	0.170
>1.0	27 (29.35)	3 (21.43)		87 (41.04)		
Capsular invasion						
Yes	65 (70.65)	9 (64.29)	0.629	155 (73.11)	0.659	0.538
No	27 (29.35)	5 (35.71)		57 (26.89)		
Multifocality						
Yes	27 (29.35)	6 (42.86)	0.309	74 (34.91)	0.345	0.547
No	65 (70.65)	8 (57.14)		138 (65.09)		
Bilaterality						
Bilateral	18 (19.57)	4 (28.57)	0.439	48 (22.64)	0.550	0.743
Unilateral	74 (80.43)	10 (71.43)		164 (77.36)		
With HT^d^						
Yes	11 (11.96)	3 (21.43)	0.329	46 (21.70)	0.046	NS^f^
No	81 (88.04)	11 (78.57)		166 (78.30)		
Thyroid nodular goiter						
Yes	55 (59.78)	8 (57.14)	0.851	114 (53.77)	0.333	0.806
No	37 (40.22)	6 (42.86)		98 (46.23)		
Lymph node metastasis						
Yes	55 (59.78)	9 (64.29)	0.898	136 (64.15)	0.562	0.895
No	33 (35.87)	5 (35.71)		70 (33.02)		
ETE^e^						
Yes	15 (16.30)	2 (14.29)	0.797	48 (22.64)	0.279	0.740
No	73 (79.35)	12 (85.71)		164 (77.36)		
Treatment of iodine-131						
Treated	28 (30.43)	2 (14.28)	0.705	78 (36.79)	0.088	0.283
Untreated	48 (52.18)	6 (42.86)		83 (39.15)		
Missing data	16 (17.39)	6 (42.86)		51 (24.06)		
T stage						
T1+T2	82 (89.13)	13 (92.86)	NS	186 (87.74)	0.730	NS^f^
T3+T4	10 (10.87)	1 (7.14)		26 (12.26)		
AJCC stage						
I+II	91 (98.91)	13 (92.86)	0.248	208 (98.11)	NS	0.276
III+IV	1 (1.09)	1 (7.14)		4 (1.89)		

a SNMTC, sporadic non-medullary thyroid carcinoma.

b pa, two affected members group vs. SNMTC.

c pb, three or more affected members group vs. SNMTC.

d HT, Hashimoto thyroiditis.

e ETE, extrathyroidal extension.

f NS, not significant.

The 106 patients were divided into the parent–offspring group and the sibling group according to their family identity. Among them, 53 (50.00%) patients belonged to the parent–offspring group, 49 (46.23%) patients to the sibling group, and 4 (3.77%) patients belonged to both two group. However, all observed clinicopathological factors between parent–offspring group, sibling group, and SNMTC did not have statistically significant difference (*p* > 0.05) (**Table 4**).

**Table 4:** Clinicopathological characteristics and prognostic factors of t parent/offspring type group and sibling type group vs. SNMTC, n (%).

Parameter	Parent/offspring type	Sibling type	*p*	SNMTC^a^	*p*a^b^	*p*b^c^
	(n = 57)	(n = 53)		(n = 212)		
Gender						
Male	15 (26.32)	8 (15.09)	0.148	56 (26.42)	0.988	0.085
Female	42 (73.68)	45 (84.91)		156 (73.58)		
Age						
<55 years	46 (80.70)	44 (83.02)	0.753	186 (87.74)	0.171	0.364
≥55 years	11 (19.30)	9 (16.98)		26 (12.26)		
Tumor size (cm)						
≤1.0	39 (68.42)	39 (73.58)	0.551	125 (58.96)	0.194	0.058
>1.0	18 (31.58)	14 (26.42)		87 (41.04)		
Capsular invasion						
Yes	38 (66.67)	38 (71.70)	0.568	155 (73.11)	0.483	0.836
No	19 (33.33)	15 (28.30)		57 (26.89)		
Multifocality						
Yes	17 (29.82)	17 (32.08)	0.799	74 (34.91)	0.472	0.698
No	40 (70.18)	36 (67.92)		138 (65.09)		
Bilaterality						
Bilateral	11 (19.30)	12 (22.64)	0.667	48 (22.64)	0.588	NS
Unilateral	46 (80.70)	41 (77.36)		164 (77.36)		
With HT^d^						
Yes	7 (12.28)	9 (16.98)	0.485	46 (21.70)	0.113	0.449
No	50 (87.72)	44 (83.02)		166 (78.30)		
Thyroid nodular goiter						
Yes	32 (56.14)	33 (62.26)	0.514	114 (53.77)	0.750	0.266
No	25 (43.86)	20 (37.74)		98 (46.23)		
Lymph node metastasis						
Yes	36 (63.16)	32 (60.38)	0.764	136 (64.15)	0.688	0.443
No	21 (36.84)	21 (39.62)		70 (33.02)		
ETE^e^						
Yes	9 (15.79)	9 (16.98)	0.866	48 (22.64)	0.261	0.370
No	48 (84.21)	44 (83.02)		164 (77.36)		
Treatment of iodine-131						
Treated	16 (28.07)	14 (26.42)	0.896	78 (36.79)	0.154	0.127
Untreated	28 (49.12)	26 (49.56)		83 (39.15)		
Missing data	13 (22.81)	13 (24.52)		51 (24.06)		
T stage						
T1 + T2	52 (91.23)	46 (86.79)	0.456	186 (87.74)	0.464	0.852
T3 + T4	5 (8.77)	7 (13.21)		26 (12.26)		
AJCC stage						
I + II	56 (98.25)	52 (98.11)	0.959	208 (98.11)	NS^f^	NS^f^
III + IV	1 (1.75)	1 (1.89)		4 (1.89)		

a SNMTC, sporadic non-medullary thyroid carcinoma.

b pa, parent–offspring group vs. SNMTC.

c pb, sibling group vs. SNMTC.

d HT, Hashimoto thyroiditis.

e ETE, extrathyroidal extension.

f NS, not significant.

### *BRAF* V600E Mutation and *TERT* Promoter Mutation of FNMTC and SNMTC

**Figure 1:**
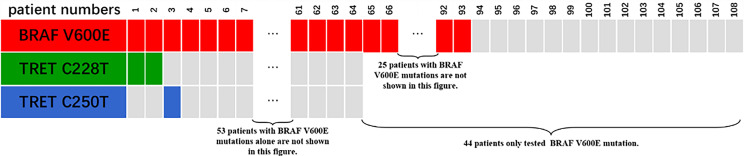
Genetic test results of 49 cases of FNMTC patients. Forty-nine of 106 included FNMTC patients were tested for *BRAF* V600E mutations, and 28 were also tested for *TERT* promoter mutations, while only 1 (3.57%) of the FNMTC patients tested positive for *TERT* promoter mutation (C228T) and 39 (79.59%) for *BRAF* V600E mutation.

**Figure 2:**
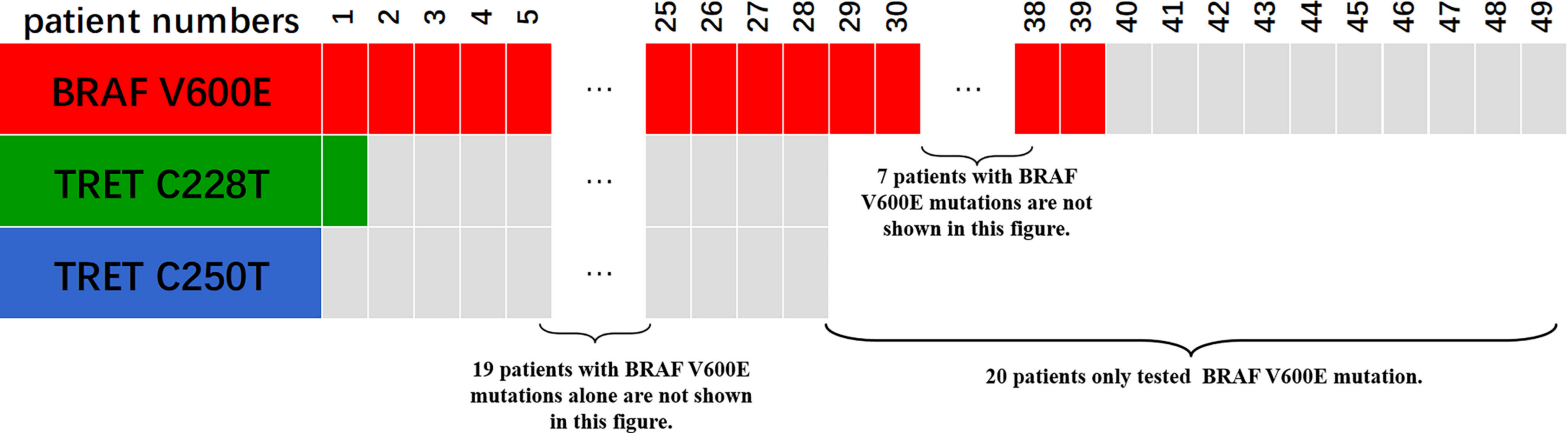
Genetic test results of 108 cases of SNMTC patients. One hundred eight patients were tested for *BRAF* V600E mutations, and 64 were also tested for *TERT* promoter in the SNMTC group, while 3 (4.69%) of the SNMTC patients tested positive for *TERT* promoter mutation (2 were C228T mutations, and 1 was C250T mutation) and 93 (86.11%) for *BRAF* V600E mutation.

Only 49 of the 106 included FNMTC patients were tested for *BRAF* V600E mutations, and 28 were also tested for *TERT* promoter mutations (**Figure 1**). Of the 212 cases in the SNMTC group, 108 patients were tested for *BRAF* V600E mutations, and 64 were also tested for *TERT* promoter (**Figure 2**).

Only one (3.57%) of the FNMTC patients tested for *TERT* promoter mutation was positive (C228T), and *BRAF* V600E mutation was positive in 39 (79.59%). Compared with SNMTC patients, there was no significance difference in BRAF V600E mutation and TERT promoter mutation between FNMTC and SNMTC (*p* > 0.05) (**Tables 5**, **6**).

**Table 5:** *BRAF* V600E mutations in FNMTC and SNMTC, n (%).

Parameter	FNMTC^a^	SNMTC^b^	*p*
	(n = 49)	(n = 108)	
*BRAF* V600E			
Wild type	10 (20.41)	15 (13.89)	0.301
Mutation	39 (79.59)	93 (86.11)	

a FNMTC, familial non-medullary thyroid carcinoma.

b SNMTC, sporadic non-medullary thyroid carcinoma.

**Table 6:** *TRET* promoter mutations in FNMTC and SNMTC, n (%).

Parameter	FNMTC^a^	SNMTC^b^	*p*
	(n = 28)	(n = 64)	
*TRET* promoter			
Wild type	27 (96.43)	61 (95.31)	NS^c^
Mutation	1 (3.57)	3 (4.69)	

a FNMTC, familial non-medullary thyroid carcinoma.

b SNMTC, sporadic non-medullary thyroid carcinoma.

c NS, not significant.

We also analyzed *BRAF* V600E mutation and clinicopathological characteristics in FNMTC and SNMTC and found that *BRAF* mutation in this study was not associated with gender, age, tumor size, inclusion, bilateral growth, thyroid nodular goiter, multifocal, lymph node metastasis, thyroid extradiffusion, iodine 131 treatment, T stage, and AJCC stage (*p* > 0.05) (**Tables 7**, **8**).

**Table 7:** Relationship of *BRAF* V600E with clinicopathological factors of FNMTC, n (%).

Parameter	BRAF V600E		*p*
	Mutation (n = 39)	Wild type (n = 10)	
Gender			
Male	10 (25.64)	0 (0.00)	0.097
Female	29 (74.35)	10 (100.00)	
Age			
<55 years	30 (76.92)	8 (80.00)	NS^c^
≥55 years	9 (23.07)	2 (20.00)	
Tumor size (cm)			
≤1.0	21 (53.85)	7 (70.00)	0.482
>1.0	18 (46.15)	3 (30.00)	
Capsular invasion			
Yes	26 (66.67)	8 (80.00)	0.702
No	13 (33.33)	2 (20.00)	
Multifocality			
Yes	14 (35.90)	1 (10.00)	0.145
No	25 (64.10)	9 (90.00)	
Bilaterality			
Bilateral	8 (20.51)	1 (10.00)	0.663
Unilateral	31 (79.49)	9 (90.00)	
With HT^a^			
Yes	3 (7.69)	2 (20.00)	0.267
No	36 (92.31)	8 (80.00)	
Thyroid nodular goiter			
Yes	26 (66.67)	6 (60.00)	0.721
No	13 (33.33)	4 (0.40.00)	
Lymph node metastasis			
Yes	24 (61.54)	7 (70.00)	0.726
No	15 (38.46)	3 (30.00)	
ETE^b^			
Yes	9 (23.08)	1 (10.00)	0.663
No	30 (76.92)	9 (90.00)	
Treatment status of iodine-131			
Treated	11 (28.21)	6 (60.00)	0.27
Untreated	20 (51.28)	4 (40.00)	
Missing data	8 (20.51)	0 (0.00)	
T stage			
T1 + T2	33 (84.62)	9 (90.00)	NS
T3 + T4	6 (15.38)	1 (10.00)	
AJCC stage			
I + II	38 (97.44)	10 (100)	NS
III + IV	1 (2.56)	0 (0.00)	

a HT, Hashimoto thyroiditis.

b ETE, extrathyroidal extension.

c NS, not significant.

**Table 8:** Relationship of *BRAF* V600E with clinicopathological factors of SNMTC, n (%).

Parameter	BRAFV600E		*p*
	Mutation (n = 93)	Wild type (n = 15)	
Gender			
Male	27 (29.03)	6 (40.00)	0.392
Female	66 (70.96)	9 (60.00)	
Age			
<55 years	88 (94.62)	15 (100.00)	NS^c^
≥55 years	5 (5.37)	0 (0.00)	
Tumor size (cm)			
≤1.0	55 (59.13)	8 (53.33)	0.672
>1.0	38 (40.86)	7 (46.66)	
Capsular invasion			
Yes	64 (68.81)	11 (73.33)	NS
No	29 (31.18)	4 (26.66)	
Multifocality			
Yes	32 (34.40)	5 (33.33)	0.935
No	61 (65.59)	10 (66.66)	
Bilaterality			
Bilateral	22 (23.65)	3 (20.00)	NS
Unilateral	71 (76.34)	12 (80.00)	
With HT^a^			
Yes	21 (22.58)	2 (13.33)	0.417
No	72 (77.41)	13 (86.66)	
Thyroid nodular goiter			
Yes	54 (58.06)	8 (53.33)	0.731
No	39 (41.93)	7 (46.66)	
Lymph node metastasis			
Yes	63 (67.74)	9 (60.00)	0.555
No	30 (32.25)	6 (40.00)	
ETE^b^			
Yes	18 (19.35)	4 (26.66)	0.502
No	75 (80.64)	11 (73.33)	
Treatment status of iodine-131			
Treated	33 (35.48)	7 (46.66)	0.725
Untreated	35 (37.63)	6 (40)	
Missing data	25 (26.88)	2 (13.33)	
T stage			
T1 + T2	82 (88.17)	11 (73.33)	0.218
T3 + T4	11 (11.82)	4 (26.66)	
AJCC stage			
I + II	93 (100.00)	15 (100.00)	NS
III + IV	0 (0.00)	0 (0.00)	

a HT, Hashimoto thyroiditis.

b ETE, extrathyroidal extension.

c NS, not significant.

## Discussion

With up to 10% of NMTC (3–6), FNMTC was generally considered as a separate malignancy in thyroid cancer with controversial clinical behavior and prognosis. At present, several studies have shown that FNMTC was more aggressive and had a worse prognosis than SNMTC (5, 7–11), but some studies have shown that there was no significant difference in biological behavior or prognosis between the two diseases (12–14). Since lots of medical records did not demonstrate whether the patient had a family history, according to the data we collected, FNMTC cases accounted for nearly 2% in our research, lower than those of former studies. We decided to randomly selected 212 patients without family history to match 1:2 ratio. However, due to limited sample data, age, gender, and tumor stage were not matched.

Cao et al. (10) and Ito et al. (15) found that FNMTC had higher multiple foci incidence, and Cao’s study also showed that it has a higher bilateral incidence. Zhang et al. found a higher incidence of lymph nodes metastases of FNMTC (5). Meanwhile, compared with SNMTC, FNMTC had a higher recurrence rate and mortality (7, 10, 21, 22). However, Moses et al. (13) found that there were no significant differences between FNMTC and SNMTC in multiple foci, bilateral incidence, and the rate of lateral lymph node metastasis; Ito et al. and Zhang et al. also proved that there was no significant difference in recurrence mortality between the two types.

Recently, with the extensive use of thyroid ultrasound technology and FNA in China, TC was found and treated at an early stage. In our study, we compared some of the invasion-related risk factors, including gender, age, tumor size, capsular invasion, bilateral growth, with thyroid nodular goiter or not, multifocality, lymph node metastasis, extrathyroidal extension, iodine 131 treatment, T stage, and AJCC stage, for FNMTC and SNMTC. We found that the tumor size of FNMTC is significantly smaller than SNMTC (*p* = 0.020), while there was no significant difference in the incidence of other factors (*p* > 0.05). We also compared the recurrence rate and mortality between FNMTC and SNMTC; same with Moses et al. and Ito et al, there was no significant difference between these two types (*p* > 0.05). One hundred four FNMTC patients (98.11%) and 208 (98.11%) SNMTC patients belong to AJCC I and II stage. These results suggested that the aggressiveness of FNMTC was not significantly different from that of SNMTC, while the average tumor size of our data (0.96 and 1.15 cm) was smaller than that of Park et al. (11) (1.2 and 1.4 cm), Robenshtok et al. (14) (1.78 and 2.02 cm), and Uchino et al. (22) (1.98 and 2.05 cm). These might mean that NMTC was detected early with thyroid ultrasound technology and FNA, and FNMTC was detected earlier than SNMTC.

Another study by Zhang et al. (7) showed that the subgroup with three or more members in the family affected with the disease was more invasive than the subgroup with only two members infected. Park et al. (11) also believed that the parent–child group was more aggressive than the sibling group, while Cao et al. (10) came to a different conclusion. Therefore, we also compared the invasive differences between different subgroups and SNMTC patients. Similar to Cao et al. (10), SNMTC only showed more significant opportunity with Hashimoto thyroiditis than two affected members group (*p* = 0.046). This could be explained by the research conclusions of Zeng et al. and Azizi et al.; they suggested that Hashimoto’s thyroiditis played a protective factor in NMTC (23, 24). Our results showed that there were no significant statistical differences in other factors between each subgroup and between each subgroup and SNMTC (*p* > 0.05), i.e., the aggressiveness of each subgroup was no more than that of SNMTC. However, the number of patients from three or more person families was relatively small, and further studies are needed to compare.

These results might be caused by the early detection and intervention of FNMTC when the tumor size of FNMTC remains small. We speculated that due to the extensive application of ultrasound and FNA in China and the active monitoring of asymptomatic first-degree relatives of patients with FNMTC, it can be detected early.

Studies have shown that *BRAF* V600E mutation was associated with large tumor size, thyroid capsule invasion, extraglandular invasion, lymph node metastasis, and high AJCC stage in PTC patients (25, 26). The *TERT* promoter mutation was related to the age of PTC patients, the maximum tumor diameter, the status of thyroid capsular invasion, and AJCC stage, according to Jin et al. (27) and Alzahrani et al. (28). Unfortunately, similar results were not obtained in our current study, which may be related to the small sample size of our genetic testing, and *TERT* was not analyzed due to the small number of mutations. Moreover, in one of our previous studies (29), we found that *BRAF* V600E mutations were mainly associated with recurrence and metastasis, which also confirmed that BRAF could be used as an indicator to evaluate the aggressiveness of NMTC. Therefore, *BRAF* V600E and *TERT* promoter mutations were also used as the evaluation indicators of FNMTC aggressiveness and was compared with SNMTC; the difference between them was not statistically significant (*p* > 0.05). This also indicated that there was no difference in clinicopathological invasiveness between FNMTC and SNMTC from a genetic perspective, while researchers generally believed that *BRAF* V600E can drive the growth of PTC through the mitogen-activated protein kinase (MAPK) pathway, and *TERT* promoter mutation might have a similar effect (25–30).

There were some limitations in our study. First, only 106 patients with FNMTC were finally included in the comparison of clinicopathological features. In the subgroup analysis, the case number of some subgroups was small, so the comparison could not be made in the statistical analysis, and the comparison results were meaningless. Furthermore, the biological behavior of NMTC patients was less aggressive, and the prognosis was better than that of other malignant tumors, so long-term follow-up is needed to explore the recurrence and metastasis. The average follow-up time of this study was 3–4 years, and some patients still need to be followed up to evaluate the recurrence and metastasis. All the patients in this study received surgical treatment shortly after the diagnosis of NMTC by FNA, so it was impossible to compare the biological behavior and prognosis differences between early and late intervention of FNMTC in this study, which need further exploration and study by follow-up researchers. 

## Conclusion

In summary, by comparing the biological behavior, prognosis, and molecular level of FNMTC and SNMTC, we draw conclusion that the biological behavior and prognosis of FNMTC were no more aggressive and worse than SNMTC, and *BRAF* V600E and *TERT* also provided a genetic explanation for this conclusion. We speculated that it might be the early detection of FNMTC with increasing emphasis on the family history that led to the result of a significantly smaller mean tumor size of FNMTC than that of SNMTC. 

## Data Availability Statement

The original contributions presented in the study are included in the article/supplementary material. Further inquiries can be directed to the corresponding author. 

## Ethics Statement

All procedures in this study were reviewed and approved by the Clinical Trial and Biomedical Ethics Committee of West China Hospital of Sichuan University. 

## Author Contributions

TY was responsible for the experimental design, experimental data collection, analysis and drafting of papers. LH assisted in data collection and analysis. CC was mainly responsible for genetic testing of samples. HL was responsible for experimental guidance and assisted in data collection. YJ was responsible for the overall experimental design, experimental guidance, and important modification of papers. All authors contributed to the article and approved the submitted version.

## Funding

This work is supported by Chengdu Science and Technology Program (No. 2019-YF05-00324-SN) and 1·3·5 Project for Disciplines of Excellence–Clinical Research Incubation Project, West China Hospital, Sichuan University (No. 2020HXFH024).

## Conflict of Interest

The authors declare that the research was conducted in the absence of any commercial or financial relationships that could be construed as a potential conflict of interest.

## Publisher’s Note

All claims expressed in this article are solely those of the authors and do not necessarily represent those of their affiliated organizations, or those of the publisher, the editors and the reviewers. Any product that may be evaluated in this article, or claim that may be made by its manufacturer, is not guaranteed or endorsed by the publisher.
